# Development of a novel green coating process with laser

**DOI:** 10.1038/s41598-022-10351-4

**Published:** 2022-04-15

**Authors:** Chongliang Zhong, Gerhard Backes, Lukas Martin Johann, Jochen Kittel, Thomas Schopphoven, Wolfgang Küppers

**Affiliations:** 1 Institute for Laser Technology, Fraunhofer ILT, Steinbachstr. 15, 52074 Aachen, Germany; 2 RWTH Aachen University DAP, Digital Additive Production, 52074 Aachen, Germany

**Keywords:** Engineering, Materials science

## Abstract

Corrosion causes costs of about 3–4% of each country’s gross domestic product, and due to the climate change, the corrosion rates on infrastructure are likely to increase furtherly in the future^1,2^. For corrosion protection, hard chrome plating (HCP) is commercially used since the early 20th^3^. Yet the biggest drawback concerns environmental protection, since toxic and carcinogenic hexavalent chromium Cr^6+^ is used. As an alternative, thermal spray (TS) is increasingly used since the last 20 years. Nevertheless, the coatings are technologically constrained in regard to high porosity, low material efficiency and poor bonding to the base material. Therefore, the demand for an environmental friendly and economical process that produces high-quality coatings is increasingly coming into the research focus. With laser, dense, metallurgically bonded and therefore well-adhering coatings with high material efficiency of > 90% can be produced from a large number of metallic alloys without the need to use environmentally harmful chemicals or noise emissions. However, the typical area coating rate of < 0.4 m^2^/h is significantly lower than that of TS of about 10 m^2^/h^4,5^, and is too small for coating large-scale components. In this regard, a novel coating process with laser was developed in this work. By decoupling the melding of powder from the coating speed, the coating speed of < 2 m/min in conventional laser coating can be increased to > 500 m/min. Defect-free layers that metallurgically bond to the base material with a thickness of 50–250 µm and a material efficiency of > 90% can be achieved. According to the results, an area coating rate up to 20 m^2^/h is achievable. The pioneer work of applications in offshore and automobile sectors show, this process is already providing beneficial for the industry.

## Introduction

Corrosion causes costs of about 3–4% of each country’s gross domestic product. The corrosion damage in 2013 is estimated to be in the magnitude of US$ 2.5 trillion globally^1,2^. Due to the climate change, in many regions, the corrosion rates on infrastructure are likely to furtherly increase in the future^3,4^. By increasing the component life with appropriate protective layers, sustainability can be significantly increased over the entire life cycle of a component, and immense cost savings potential can be tapped^5^. Protective layers contribute as well to protect people and nature, for example fine dust emissions due to the frictional wear of car brake discs can be significantly reduced^6–8^. In the production of corrosion and wear-resistant coatings, thin metallic protective layers with layer thicknesses in the range from 50 to 250 µm, which are applied using hard chrome plating (5–250 µm) or thermal spray processes (100–500 µm)^9^, are state of the art. However, these processes have decisive environmental deficits (use of environmentally harmful chemicals, noise emissions, high fuel and material consumption, etc.) as well as technological deficits (low layer adhesion, limited corrosion resistance, layer defects, etc.)^10–15^.

For corrosion protection, hard chrome plating (HCP) has been being commercially used since the early twentieth century^16^. Layers with a thickness of about 5–300 µm can be generated^9,17^, and the layers of HCP show a high corrosion resistance, a high hardness and a low friction coefficient at low cost^18,19^. In various branches such as aerospace, oil and gas, automotive, papermaking, and others, HCP is a widespread, standard process. However, the implementations of EU directives such as 1999/13/EC (VOC), 2011/65/EU (RoHS), and 2012/19/EU (WEEE) as well as the EU regulation EC 1907/2006 (REACH) to environmental protection, CO2-reduction, and energy efficiency are leading to considerable market upheavals in the future. In HCP, toxic and carcinogenic hexavalent chromium (Cr^6+^) is used, which can only be used with authorization after the so-called sunset date in September 2017. Therefore, the industry is urgently in need of an environmental friendly, productive and cost-efficient alternative process which can apply corrosion resistant alloys to various substrate materials^18–23^. Additionally, the development of new coating technologies for the wear and corrosion protection of large-scale, high quality components in the manufacturing industry is becoming more and more significant, not only from an economic but also from an ecological perspective. Coating technologies which are considered capable of replacing HCP are thermal spray technologies, especially high velocity oxygen-fuel (HVOF) and direct energy deposition (DED) with laser.

With HVOF, coatings out of a large range of materials can be applied, which feature high wear resistance at relatively low investment costs. It is increasingly used for corrosion protection since the last 20 years due to its high productivity with an area coating rate of about 10 m^2^/h^24,25^. Nevertheless, the coatings exhibit a poor bonding to the base material, since the thermally sprayed coatings are, instated of a metallurgical bonded, mechanically adhered to the substrate surface, which results in some orders lower adhesive tensile strengths compared to metallurgical bonded coatings, i.e. laser cladded^26^. Moreover, the coatings are porous^27^, and are nearly not possible to be repaired. In addition, thermal spray has a low powder efficiency of only about 50%^28^. Furthermore, the coatings reveal a lamellar structure^29^. The lamellar boundaries act as corrosion paths and the detachment of the lamellae is high in harsh corrosion conditions^30^. Regarding these, HVOF coatings are technologically constrained as an alternative for HCP for corrosion protection.

DED with laser can be used to produce dense, metallurgically bonded and therefore well-adhering coatings from many metallic alloys without the need to use environmentally harmful chemicals or noise emissions. By applying precision powder feeding, a material efficiency of more than 90% can be achieved. Here, a molten pool is created on the surface of the substrate by laser radiation. At the same time, an additional material is injected into the molten pool, where it melts completely. Due to the precise spatial and temporal controllability of the heat input by using light as an energy source in combination with a defined material application, coatings with desired layer properties, such as corrosion resistance, can be achieved. Nevertheless, in the production of wear and corrosion protection layers, DED is only applied in limited and specific applications. The main reason is, the typical surface rates of < 0.4 m^2^/h are too low to be economically competitive to HCP and HVOF and are far too small for coating large-scale components. It is worth mentioning, by applying lateral powder feeding with combination of a high laser power up to 20 kW, the area coating rate can be significantly increased. However, the significant deficits in such a process are technologically constrained in regard to the large energy input, which results in an undesirable effect of the base material and a low dimensional accuracy due to deformation. Furthermore, the material efficiency is low, normally < 50% and the achievable layer thicknesses of > 500 µm is commonly too large for the wear and corrosion protection in terms of material use and post-processing effort compared to required layer thicknesses of up to 250 µm. Thus, though a high coating rate, the applications of such a process are very limited. Therefore, we concentrate here only on coaxial powder feeding. The target of our work is, with maintenance of the aforementioned coating qualities and a high material efficiency, to increase the area coating rate to more than 10 m^2^/h, which is comparable to that with HVOF. Additionally, the thin layers in the range of 50–250 µm should be achieved.

## Theory and methods

The area coating rate by applying DED with laser radiation can be calculated as follows:

1 $$ R_{c} = v \cdot d_{L} \cdot \left( {1 - \varepsilon } \right) $$

where $d_{L}$ is the width/diameter of laser spot, $v$ the coating speed, and $\varepsilon$ the overlap rate, the percentage of overlap between two tracks [%].

**Figure 1:**
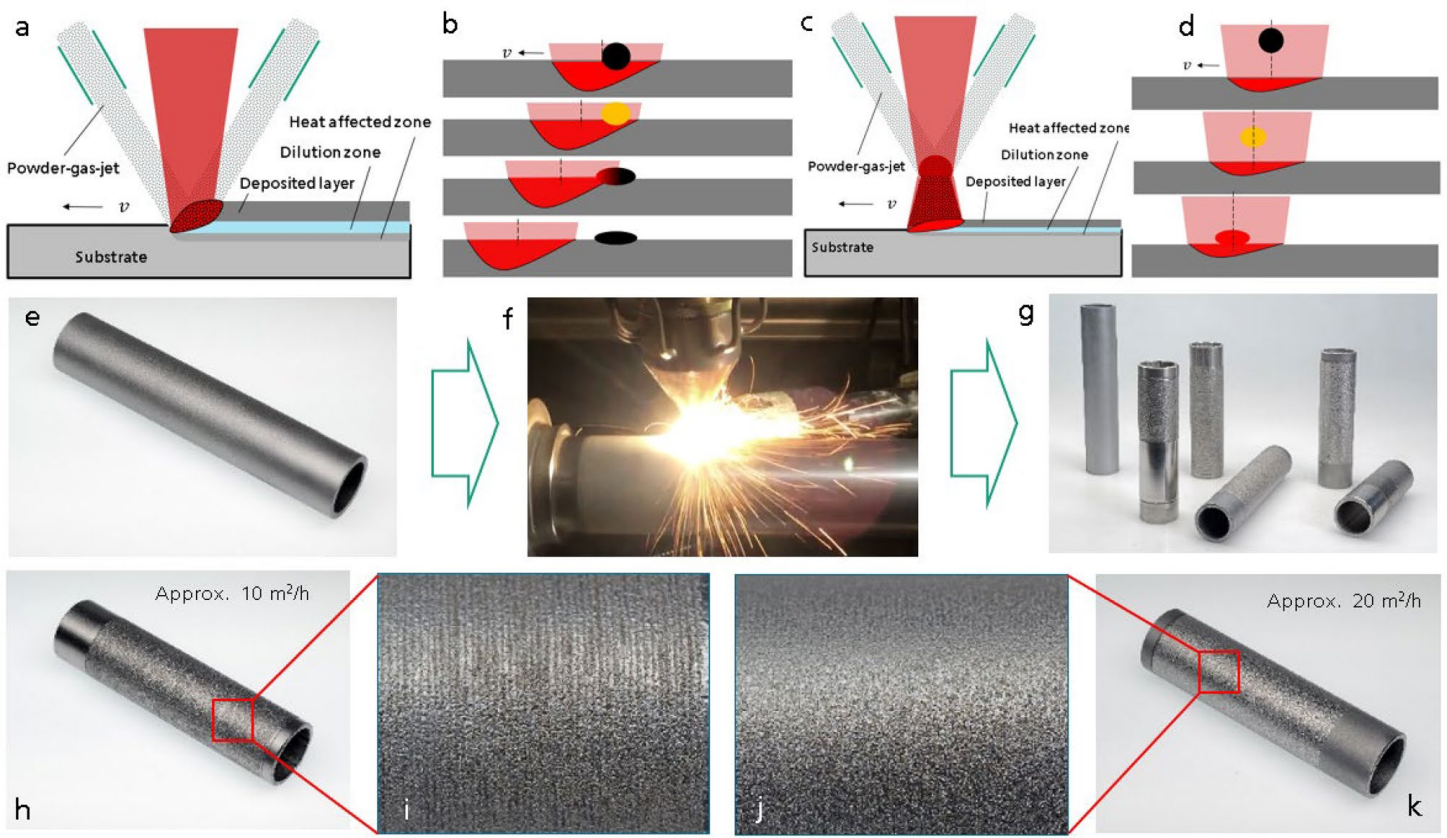
Coating with laser. (**a**,**b**) The principle of direct energy deposition (DED) with laser. The powder is injected by a powder feeding nozzle into the molten pool generated by a laser beam. Whether the powder can be molten depends strongly on the laser power and the coating speed, where the latter decides mainly the lifetime of the molten pool. (**c**,**d**) The principle for the coating process of this work. The focus of the powder-gas-jet is positioned above the surface of the substrate and the powder is injected into the laser beam. By this, the dependency of the powder melting from the coating speed is neglected—instead of melting in the molten pool, the powder is molten before it comes into the molten pool. Due to overcoming this limit, a significant increase of the coating speed can be realized, which is a positive impact on productivity improvement. (**e**–**g**) The coating process: the substrate (E355) surface is firstly sand blasted and degreased using acetone before it is cladded with Inconel 625 (IN625). An area coating rate up to about 20 m^2^/h has been achieved. (**h**,**i**) An overview of a coated tube with an area coating rate of about 10 m^2^/h and a detailed view for the coated surface. (**j**,**k**) An overview of a coated tube with an area coating rate of about 20 m^2^/h and a detailed view for the coated surface. For all the presented results, IN625 powder is used as additive material and steel E355 as base material.

With a constant overlap rate, the area coating rate depends on the size of laser spot $d_{L}$ and the coating speed $v$. As discussed above, by increasing the laser spot, namely by applying lateral powder feeding with combination of a high laser power, is not the proper approach. The possible solution for achieving the mentioned targets of our work is therefore the increase of the coating speed. The coating speed in DED with laser is normally < 2 m/min. The problem is, due to the principle of this process, schematically illustrated in Fig. 1, the achievable coating speed is restricted.

To understand this, the lifetime of the molten pool $t_{1}$ should be used, which can be roughly defined by

2 $$ t_{1} = d_{L} /v, $$

The lifetime of the molten pool $$t_{1}$$ defines the longest duration, a powder particle stays in the molten pool. Supposing that a powder particle needs a time of $$t_{2}$$ to melt, $$t_{1}$$ must be larger than $$ t_{2}$$ to ensure the melting. According to the formula, $$t_{1}$$ is inversely proportional to the coating speed $$ v$$; thus, an excessive coating speed results in an insufficient lifetime of the molten pool $$ t_{1}$$, namely $$t_{1}$$ < $$t_{2}$$. Consequently, no qualified coatings can be generated since the complete melting of powder particles, which is the main prerequisite for a sound process, cannot be ensured.

Here we see, with a constant laser spot, the melting of the powder particles depends strongly on the coating speed—an arbitrary increase of the coating speed is therefore not possible. In DED, the melting of the powder is coupled to the coating speed. Because the coating speed is limited, it is not possible to realize a significant improvement of the area coating rate. In the practice, the typical coating speed lies normally in the range of 0.5- 2 m/min, by which an area coating rate of up to ca. 0.4 m^2^/h can be achieved.

For a significant increase of the area coating rate, the coating speed must be significantly increased. Our approach, therefore, is to decouple the melting of powder from the coating speed—making both independently. In order to achieve this, we shift the focus of the powder-gas-jet and position it over the substrate surface, as shown in Fig. 1c—there is a certain distance between the powder focus and the substrate surface. The target is to melt the powder completely before it reaches the molten pool.

In this case, instead of melting in the molten pool in conventional DED, the coating material is melted in by traveling through the laser beam and comes into the molten pool in the liquid state. Therefore, whether the powder can be melted is no longer dependence on the life time of the molten pool ${t}_{1}$; thus, the life time of the molten pool ${t}_{1}$ not anymore restricts the coating speed. Since the powder-gas-jet moves together with the process head, the melting of powder is separated from the coating speed. By this way, a significant increase of the coating speed can be achieved.

In this coating process, the time needed for melting the powder depends mainly on the following three factors: (a) the gain size of the powder, (b) the duration that the powder particles stay in the laser beam and c) the laser intensity.

- To shorten the time for melting the powder, powder with finer grain size should be used, for example 20–50 µm. In conventional DED, powder with a nominal grain size of 45–90 µm, or even 90–150 µm is very often applied.
- A sufficient reaction time between the laser beam and the powder is necessary for a complete melt of the powder. The duration that the powder stays in the laser beam can be controlled by regulating the particle injection speed and/or by adjusting the distance between the substrate surface and the focus of the powder-gas-jet.
- For favoring the melting process, a high laser intensity is necessary. The laser intensity is defined by the quotient of the laser powder and the cross-sectional area of the laser beam and it represents the energy density. A high intensity can be achieved by both increasing the laser power and/or decreasing the cross-sectional area of the laser beam—the highest intensity is achieved at the focus plane.

For a sound process, there are further aspects needing attention. To use the high laser intensity sufficiently and to minimize the heat effect on the base material, the majority of the laser energy should be absorbed by the powder. This can be achieved by increase the powder flux density, which is defined by the quotient of the powder mass flow rate and the cross-section area of the powder-gas-jet. Obviously, the highest powder flux density appears at the focus plane of the powder-gas-jet. A coaxial powder feeding nozzle with a conical powder-gas-jet enables a high powder flux density.

Another essential difference of this novel coating approach in comparison to conventional DED is the mechanism of energy absorption. In DED, most of the laser energy is absorbed by the substrate for generating a molten pool, which provides sufficient energy for melting the powder particles that come into it. In this process, the energy from laser is firstly transferred to the base material and concentrated in the molten pool. This energy will be afterwards transferred to the coating material for melting it. Differently, in the process of this work, the powder particles are melted directly by the energy of the laser beam. This results in a higher energy efficiency. Additionally, the heat effect on the base material can be minimized. By this, it is possible to coat components out of sensitive materials. Moreover, the minimized heat effect additionally enables the pairing of immiscible materials, since the formation of the brittle intermetallic phases that make it impossible for pairing these materials can be avoided.

For ensuring a sound metallurgical bonding, the surface of the substrate locally must be transferred to the liquid phase. Because this allows the coating material and the based material to combined in the same aggregate state. In order to minimize the process influence on the substrate, the target is to keep the melt depth as shallow as possible. In this process, instead of injecting the powder into the melting pool primarily in solid state as in DED, the coating material is injected in liquid state. By this, the molten material is instantaneously mixed together with the base material and solidified, which ensures a low melting depth and the physical properties of the coating, they are for example wear- and corrosion resistance, as well as the ductility.

- Therefore, a small portion of laser power must can be transmitted on the surface of the base material. Because of this, the powder flux density is not allowed to be arbitrarily increased, otherwise the laser energy transmitted to the substrate is not sufficient anymore for ensuring a metallurgical bonding.
- For ensuring the melting of the outermost surface of the substrate, the higher the coating speed, the higher laser intensity is generally needed. Nevertheless, as discussed above, the amount of the transmitted intensity laser energy on the substrate surface must be limited since the heat effect on the base material should be kept as small as possible and the energy efficiency as high as possible.

## Experiments and results

For investigating our idea, an experimental setup has been designed, experiments have been conducted and the results are evaluated. The results show, by this approach, the powder can be melted before it comes into the molten pool, which indicates, the melting of powder can be decoupled from the coating speed.

The experimental setup consists mainly a high-power laser source, a powder feeder, a processing optic, a powder feeding nozzle, a 3-axel handling system and a rotary handling. In order to provide enough energy, a high-power laser source with a maximal output of 12 kW is used. For the high powder flow rates, an adapted powder feeding nozzle is used. To obtain a large laser spot, a zoom-optic is integrated. For achieving an extreme high speed of up to 1000 m/min, a high-speed rotary handling is used. The process head that consists of the processing optic and the powder feeding nozzle is mounted to the 3-axel handling system, which is NC-controlled.

The powder is fed by a powder feeder by using Argon (Ar). The powder is firstly fed into a separator before it comes into the powder feeding nozzle, through which a homogeneous powder-gas-jet is generated. For the experiments, Ar is as well used as shielding gas to protect the process zone from the atmosphere.

The laser beam generated by the laser source LDF 100–12,000 is guided into the processing optics via a light fiber with a diameter of 1 mm. After coupling, the laser beam is directed to the collimating lens. Through a focusing lens, the laser beam is then focused on the substrate surface. Via the beam path, the process can be observed coaxially on a monitor by a CCD camera.

For the experiments, we used IN625 as filler material and steel E355 as base material.

Based on the experimental setup, by applying this approach, the coating speed can be increased by factor of even more than 300, from 0.5–2 m/min in DED to up to approx. 600 m/min. By this, an area coating rate up to 20 m^2^/h with a layer thicknesses in the range of 50–250 µm can be achieved. Additionally, defect-free coatings which are metallurgically bonded to the base material with a material efficiency of > 90% is achievable.

**Figure 2:**
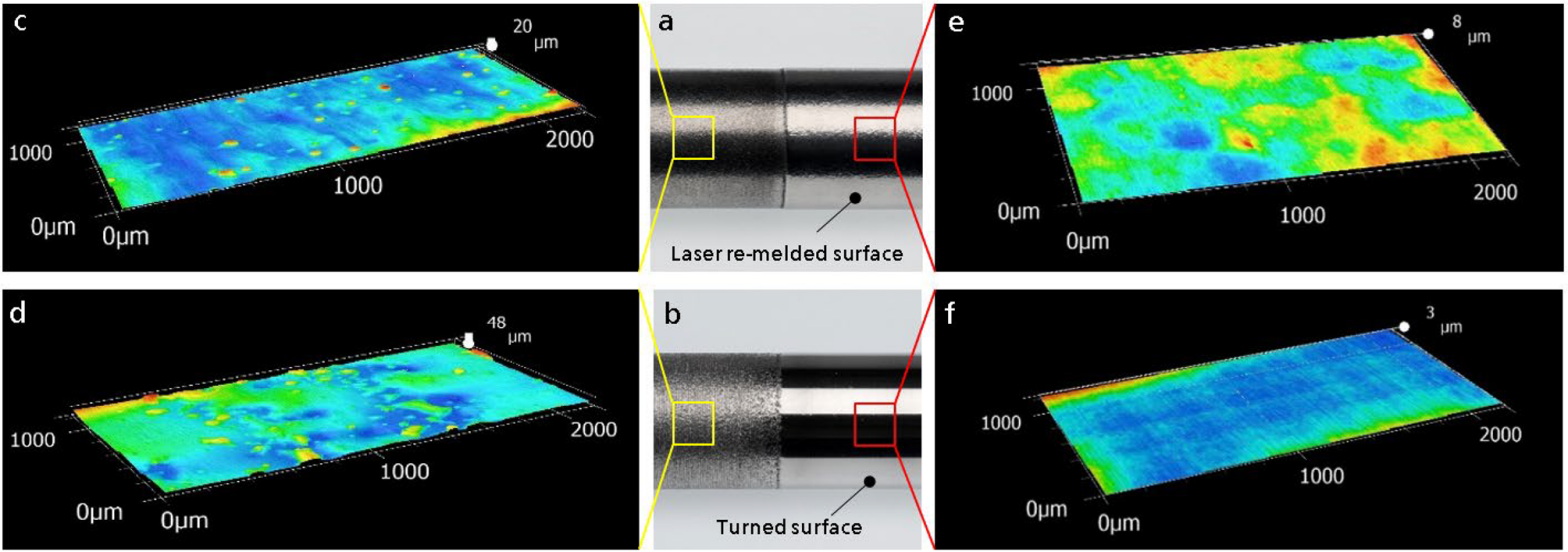
The topography of the coated and the post-processed surfaces. (**a**,**b**) Two tubes, which are coated and afterwards partially post-processed by laser re-melting (upper) and turning (lower), respectively. (**c**,**d**) The surface topography of the surfaces after coating. (**e**,**f**) The surface topography of the post-processed surfaces by laser re-melting and turning, respectively. It can be seen, the surface roughness can be essentially improved by both post-processing methods.

As shown in Fig. 1, a relative smooth surface can be directly generated. A surface roughness of S_a_ up to 10 µm and R_z_ up to 60 µm have been measured. The HCP coatings used in industrial applications have a surface roughness of about 1.2–3.2 µm in R_a_ and 6–20 µm in R_z_^23^. The achieved surface roughness is higher than that of HCP; nevertheless, this surface roughness is sufficient for applications with low requirements to the surface finish. For applications that require a smoother surface, post processing can be applied by laser re-melting or turning. After a post-processing, a comparable surface roughness to that of HCP can be achieved: through laser re-melting, the surface roughness can be reduced to S_a_ = 1 µm and R_z_ = 8 µm, and through turning, a smoother surface with S_a_ almost 0 and R_z_ = 4 µm can be achieved. As an example, the coated and the post-processed surfaces through laser re-melting and turning are presented in Fig. 2, respectively. In laser cladding, the post-processing of laser re-melting is a very convenient process, because it can be conducted just by turning off the powder and scanning the coated surface again with the laser beam. Nevertheless, with turning, a smoother surface can be obtained.

**Figure 3:**
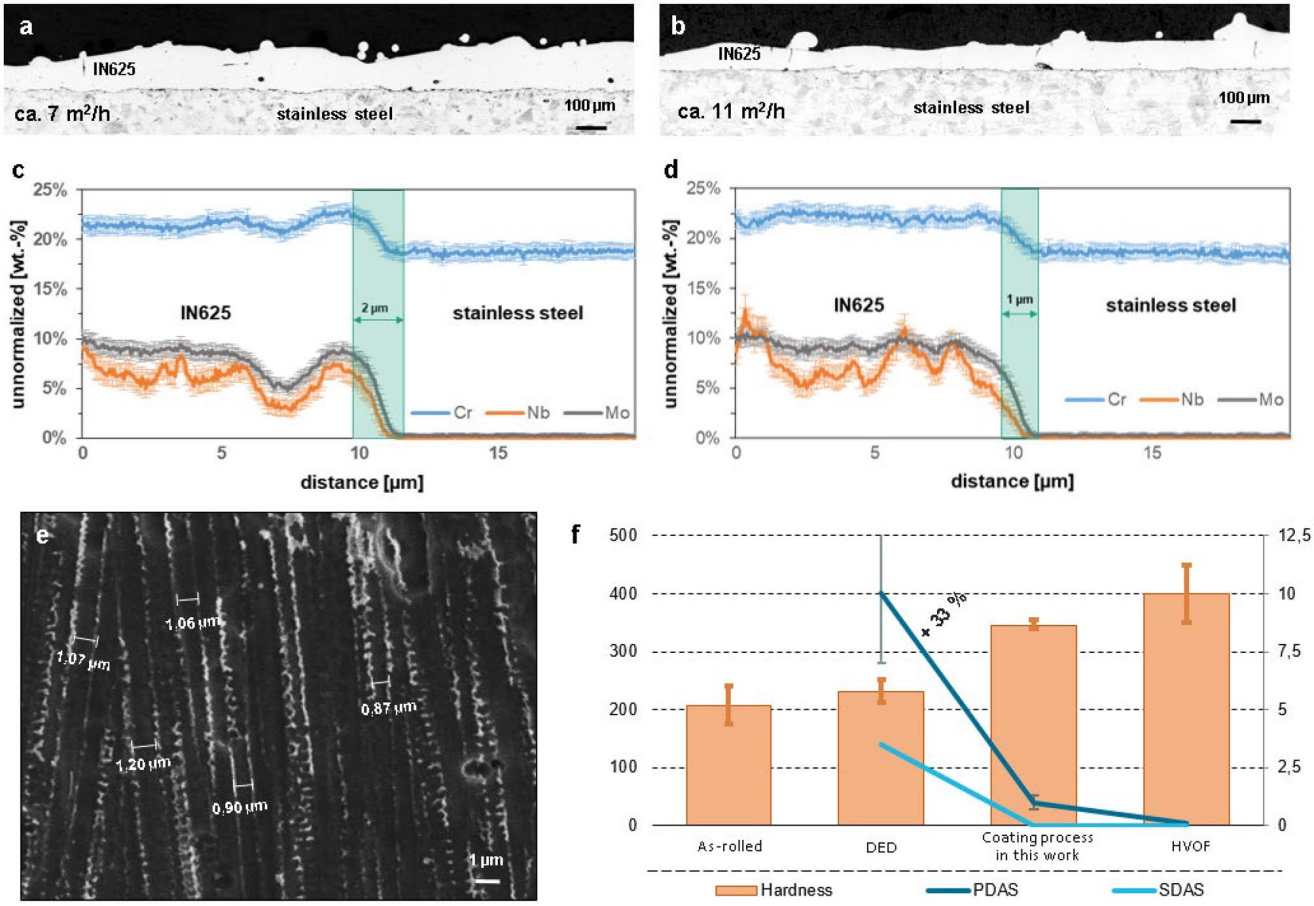
The metallurgical properties, the microstructure, and the hardness of the coatings. (**a**,**b**) The cross-sections of the coatings with an area coating rate of approx. 7 m^2^/h and 11 m^2^/h, respectively. (**c**,**d**) The results of WDS-analysis of the coatings with an area coating rate of approx. 7 m^2^/h and 11 m^2^/h, respectively. According to the results, a dilution depth of about 2 µm and 1 µm in depth are achieved for both coatings, respectively. (**e**) A scanning electron microscopy (SEM) micrograph showing the finer microstructure of the coated IN625, where some of the primary dendrite arm spacing (PDAS) are measured. (**f**) The hardness, the PDAS and the secondary dendrite arm spacing (SDAS) of IN625 produced by different processes are compared. It can be seen, the hardness of our process is significantly higher than that of raw material. It is comparable to that of HVOF and is about 33% higher than that of DED.

For wear and corrosion protection, the metallurgical properties are decisive. In Fig. 3, the cross-sections of the coated samples with an area coating rate of approx. 7 m^2^/h and 11 m^2^/h are depicted. Take the sample with an area coating rate of about 7 m^2^/h for example, the layer is defect-free and has a porosity of approx. 0.3%. Unlike HVOF coatings, there is no interconnected pores, which benefits corrosion protection.

A WDS-Analysis (Wavelength-dispersive X-ray spectroscopy) revealed, the coatings are metallurgically bonded to the substrate, which ensures a high adhesive tensile strength, shown in Fig. 3. The depth of the dilution zone is approx. 2 µm and 1 µm for coatings with an area coating rate of about 7 m^2^/h and 11 m^2^/h, respectively. With this process, a dilution of 1–2% can be achieved, which is much lower than that of DED with a dilution of about 5–10%.

Another essential effect of the tremendous increased coating speed is the change of material microstructures. This is because the microstructure is mainly influenced by the solidification process, which is in turn closely related to the cooling rate^31^. In coating with laser radiation, the cooling rate mainly depends on the coating speed, and generally: the higher the coating speed, the higher the cooling rate. With the significantly increased cooling rate a much finer microstructure can be generated, and this could increase the hardness of the material, which is typically beneficial for increasing the wear resistance. In order to verify this hypothesis, the microstructures have been analyzed and the hardness has been measured.

The analysis shows the coated layers with different area coating rates, 7–20 m^2^/h, and exhibit very similar microstructures. Since the laser coated layers normally exhibit a dendritic microstructure due to the directional solidification, the primary dendrite arm spacing (PDAS) is commonly used for describing the fineness of the microstructure quantitatively. In the developed coating process, a PDAS of approx. 1 µm is determined, which is much finer than that of DED at about 5–10 µm. By using the PDAS, the cooling rate can be calculated^32^, resulting a cooling rate between 10^5^ and 10^6^ K/s for the developed coating process. This is about two orders of magnitudes higher of that of DED, which is determined between 10^3^ and 10^4^ K/s^33,34^. This result verifies the hypothesis, and the essentially higher cooling rate distinguishes this coating process from DED. This is because, as mentioned above, the properties of a material mainly depend on the microstructure and the microstructure is essentially decided by the solidification process, which is in turn closely related to the cooling rate^31^.

The achieved average hardness of the coatings is in the range of 337 HV to 363 HV. The hardness of IN625 coated by DED is approx. 250 HV and the hardness for conventionally processed material, i.e. rolled IN625 is given with a maximum of 250 HV^35^. In comparison to these, the achieved hardness is about up to 50% higher. The increased hardness accords to the observed finer microstructures. It is worth to notice, that the achieved hardness is comparable to that of HVOF coated IN625, which is reported to be in the range of 350–450 HV^36,37^.

Now, we have demonstrated the significant increase of the area coating rate by more than two orders of magnitudes with maintained and even improved qualities. The maximum processed area coating rate even exceeds typical values of HVOF; and at the same time, the achievable material efficiency and metallurgical qualities are essentially better. This novel green coating process is the first coating alternative that is effective, economic and environmentally friendly, and it is already proving beneficial for the coating industry.

## Pilot applications in the industry

**Figure 4:**
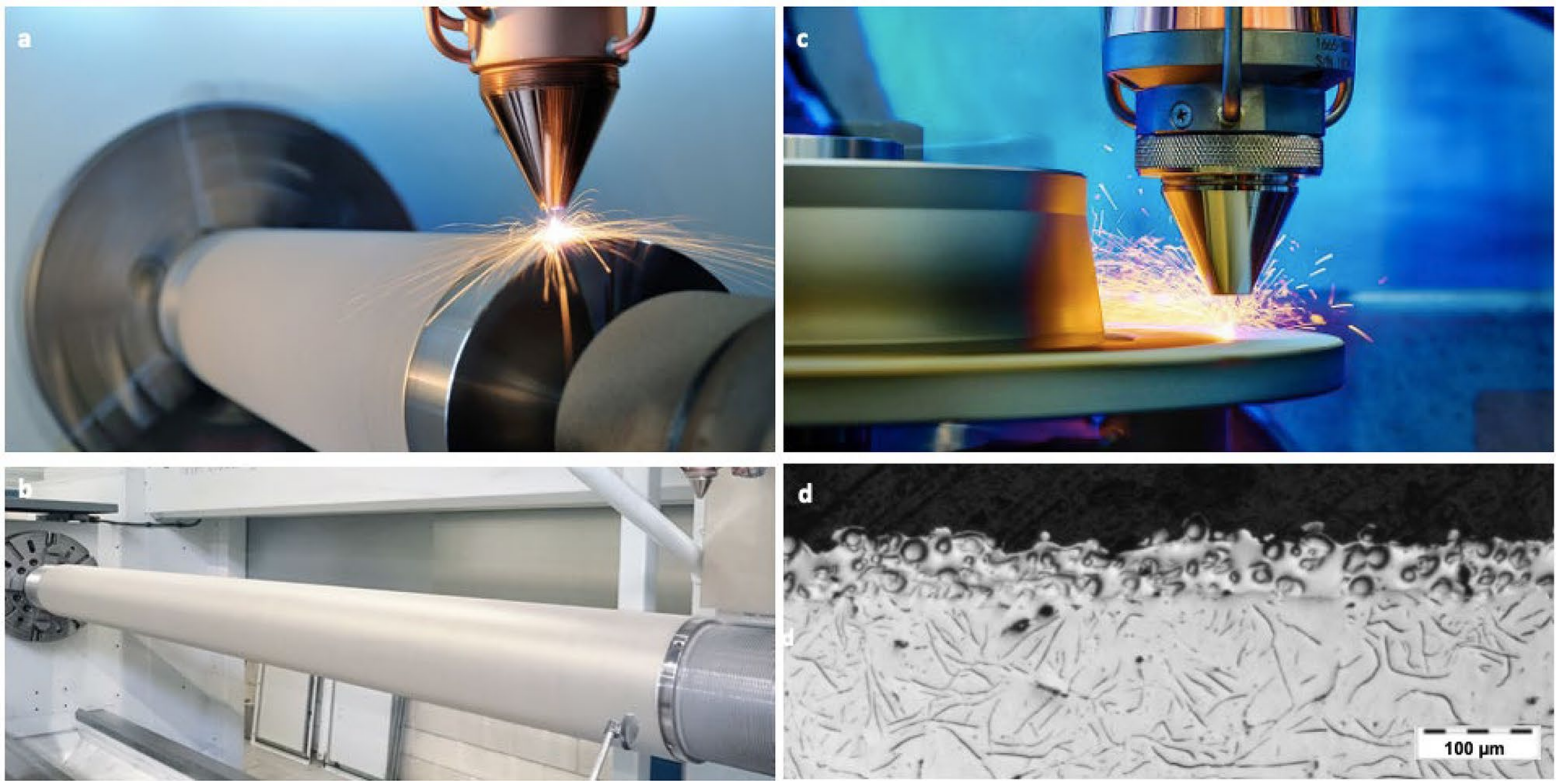
Industrial applications. (**a**) A piston rod, which is being coated. (**b**) One cladded piston rod. (**c**) Cladding of brake disc for automobile industry for wear resistance. (**d**) A cross-section of the cladded brake disc.

Some pilot applications are shown in Fig. 4. The first example is in offshore industry^38^. The company IHC Vremac Cylinders B.V. from the Netherlands produces hydraulic cylinders with piston rods up to 10 m in length. The rods are used under extreme heavy-duty conditions in excavators, offshore plants, civil engineering and heavy machinery applications and were coated by HCP for wear and corrosion protection. Due to the strict regulation of HCP since 2017, the company is searching for an alternative coating process. Though HVOF is a possible solution for hard coatings, as aforementioned, the corrosion resistance of it is poor. Additionally, the extreme high hardness results in the reduction of material ductility. Due to the low productivity, DED is not attractive for industrial production. However, this novel coating process resolves the problem. IHC Vremac Cylinders B.V. has successfully implemented this process for series production of cladding IN625 on the piston rods for corrosion and wear protection. The company has already been able to convince customers in the offshore sector about the benefits of the new coating since the process has been certified to DIN EN ISO 15614-7 by Lloyd’s Register (LR).

## Discussions

This process has a very high potential for a much wider applications in different sectors in the future, since as mentioned, a high variety of metal alloys can be processed by laser. In this regard, a high potential application is the coating of brake discs in the automobile industry^39^. Traditional brake discs are made of cast iron containing lamellar graphite phases. The advantage of this material lies in its good thermal conductivity and high thermal capacity, all for a relatively low price. The downside is however the strong propensity to corrode coupled with high material wear during service, which generates substantial emissions of fine particulate matter. To date, it has proved difficult to provide adequate protection for brake discs by means of conventional coating processes such as HCP or HVOF. The problem with this process is the poor bonding between the coating and the base material. A further disadvantage is the high resource and energy consumption. With the developed laser-based coating process, it is possible to overcome these drawbacks by applying coatings with a metallurgical bonding to the base material of the brake disc. Thanks to the minimized heat effect on the substrate surface, cast iron can be coated. In the production, a Fe-based alloy will be used as coating material for generating a buffer layer. In a further step, tungsten carbide (WC) for example is used due to its high hardness for a high wear resistance, and it is mixed with a nickel-based super alloy, which performs as the matrix. A layer thickness of approx. 60 µm with a dilution < 1% can be achieved. With this approach, the coatings do not flake and chip, but remain intact and thus provide a longer lasting and more effective corrosion protection for the component^40^. This increases service life and prevents early failure because of damage to the surface of the brake disc.

## Summary and conclusions

In summary, a novel, green laser-based coating process has been developed. The innovative strength of this process is highlighted by the principle of decoupling the melting of powder from the coating speed, more in detail:

- Instead of melting in the molten pool, like in conventional DED, the powder particles are melted completely on the way through the laser radiation. This is the main factor for enabling the decoupling the melting of powder from the melting pool.
- In conventional laser based DED, the majority of laser energy is firstly transferred to the base material for generating a molten pool, which must provide sufficient energy for melting the injected powder particles. Differently, in the process of our work, the powder is melted directly by the laser beam—the majority laser energy is absorbed by the powder.
- Because of the above point above, the heat effect on the based material can be minimized. By this, it is possible to coat sensitive materials. Moreover, the minimized heat effect enables the pairing of unconventional materials such as Fe/Ti, Fe/Al and Fe/Cu, since the formation of the brittle intermetallic phases can be avoided which make the pairing of these materials impossible. A practical example is the coating of brake disk in the automotive industry, which is not possible to be coated by conventional DED.
- In this process, the molten material is instantaneously mixed with the base material with the same aggregate state and solidified, by which good coating qualities, such as metallurgical bonding and low porosity, can be achieved. Additionally, this as well enables the physical properties of the coating, they are for example wear and corrosion resistant, as well as retaining its ductility.
- Due to the tremendous increased coating speed, the process exhibits a cooling rate in the range of 10^5^–10^6^ K/s, which is significantly higher than that of conventional DED with laser of about 10^3^–10^4^ K/s^41^. The higher cooling rate results in a finer microstructure, and therefore a higher hardness, which is beneficial for the property of wear resistance.

The results show, by applying this approach, the coating speed from 0.5 to 2 m/min in conventional DED can be increased by orders of magnitudes to about 600 m/min. As demonstrated, an area coating rate of up to 20 m^2^/h has been achieved, which exceeds the typical values of thermal spraying such as HVOF. Defect-free coatings with a layer thickness of 50–250 µm, metallurgical bond to the base material, and a material efficiency of > 90% are achieved. The applied layer thickness fulfills the requirement of corrosion and wear-resistant coatings. In compassion to conventional DED, the coatings maintained or even show improved metallurgical qualities. Additionally, a hardness increase of more than 30% is achieved as well.

This process is already proving benefits for industry, such as in the offshore and automobile sector. The application potential of this green process is enormous. It utilizes resources more efficiently, can produce high quality layers from many metallic alloys without the need to use environmentally harmful chemicals or noise emissions. Therefore, it provides an effective, economic and environmentally friendly solution for the industry. Furthermore, it could be used for coating large-scale components nowadays used without coatings. This would make it possible to produce innovative components that do not wear out during a product’s lifecycle.
